# Amelioration of Streptozotocin-Induced Diabetes in Mice with Cells Derived from Human Marrow Stromal Cells

**DOI:** 10.1371/journal.pone.0002666

**Published:** 2008-07-16

**Authors:** Min Zhao, Stephanie A. Amiel, Sanaz Ajami, Jie Jiang, Mohamed Rela, Nigel Heaton, Guo Cai Huang

**Affiliations:** 1 Diabetes Research Group, King's College London School of Medicine, London, United Kingdom; 2 Department of Haematological Medicine, King's College London School of Medicine, London, United Kingdom; 3 Institute of Liver Studies, King's College Hospital, London, United Kingdom; University of Aberdeen, United Kingdom

## Abstract

**Background:**

Pluri-potent bone marrow stromal cells (MSCs) provide an attractive opportunity to generate unlimited glucose-responsive insulin-producing cells for the treatment of diabetes. We explored the potential for human MSCs (hMSCs) to be differentiated into glucose-responsive cells through a non-viral genetic reprogramming approach.

**Methods and Findings:**

Two hMSC lines were transfected with three genes: PDX-1, NeuroD1 and Ngn3 without subsequent selection, followed by differentiation induction *in vitro* and transplantation into diabetic mice. Human MSCs expressed mRNAs of the archetypal stem cell markers: Sox2, Oct4, Nanog and CD34, and the endocrine cell markers: PDX-1, NeuroD1, Ngn3, and Nkx6.1. Following gene transfection and differentiation induction, hMSCs expressed insulin *in vitro*, but were not glucose regulated. After transplantation, hMSCs differentiated further and ∼12.5% of the grafted cells expressed insulin. The graft bearing kidneys contained mRNA of insulin and other key genes required for the functions of beta cells. Mice transplanted with manipulated hMSCs showed reduced blood glucose levels (from 18.9+/−0.75 to 7.63+/−1.63 mM). 13 of the 16 mice became normoglycaemic (6.9+/−0.64 mM), despite the failure to detect the expression of SUR1, a K^+^-ATP channel component required for regulation of insulin secretion.

**Conclusions:**

Our data confirm that hMSCs can be induced to express insulin sufficient to reduce blood glucose in a diabetic mouse model. Our triple gene approach has created cells that seem less glucose responsive in vitro but which become more efficient after transplantation. The maturation process requires further study, particularly the *in vivo* factors influencing the differentiation, in order to scale up for clinical purposes.

## Introduction

Restoring beta cell mass by islet cell transplantation in type 1 diabetes has become a realistic option for the treatment of Type 1 diabetes mellitus (T1DM) [1], although it faces problems, including a very limited supply of donor organs; the tendency for loss of islet function *in vivo* over time; toxicity of current immunosuppression regimens and risk of rejection and reoccurrence of autoimmune attack. In order to make beta cell replacement a more broadly applicable therapy, a source of surrogate glucose regulated insulin-secreting cells, preferably autologous, must be harnessed. Human MSCs, also known as colony-forming-mesenchymal cells, are readily expanded *in vitro* [2], [3] and can be derived from bone marrow as well from peripheral blood [4]. These cells contain long telomeres [5] and no telomerase activity [6], [7]. As both length of telomeres and telomerase activity are implicated in the immortality of tumour cells [8]–[10] and embryonic stem cells [10], [11], the lack of telomerase activity in hMSCs may imply reduced likelihood of tumour generation. MSCs have been shown to express both archetypal stem cell markers and early markers of differentiated tissues [12]–[15] and also have the potential to differentiate into a variety of cell types and tissues [16]–[19], including insulin-producing cells [5], [20]–[22], albeit at low level. One recent publication documented the production of insulin expressing cells sufficient to improve, although not normalise, hyperglycaemia in diabetic mice through an adeno-rat Pdx-1 recombinant virus [23]. We have used a stepwise, non-viral genetic reprogramming approach, in order to influence hMSCs to differentiate into cells with properties of the beta cell phenotype *in vitro*, in readiness for subsequent maturation into glucose-responsive insulin-producing cells *in vivo*. We have analysed the phenotype of these cells in comparison with human islet cells.

## Methods

### Cell Culture

Two human stromal cell lines (220R and 240L) [24], donated by Professor Darwin J. Prockop, Center for Gene Therapy, Tulane University, were used in this study. The cells were assigned as the 1^st^ passage on arrival at King's. The cells were cultured in α-MEM (Sigma, Dorset, UK) supplemented with 16.67% FCS at 37°C in a humid 5% CO_2_ incubator and expanded by culturing at ∼2% density (60,000/10 cm petri dish) and harvested at ∼30% to prevent the loss of multipotency. For differentiation induction study, cells at passage 4–6 were used.

### Cell proliferation assay

Cells were seeded at ∼2% density and cultured for 5 days. Portions of the cells were dissociated with trypsin/EDTA solution daily and cell number was counted. The remaining dissociated cells were centrifuged down as a pellet, processed for cryosections and stained for the Ki67 marker as described previously [25].

### Human Pdx-1, NeuroD1 and Ngn3 expression plasmids and transfection

The coding regions of the human PDX-1, NeuroD1 and Ngn3 genes were amplified respectively by RT-PCR using specific primer sets (Table 1). The genes were then respectively cloned into the expression plasmid–pIRES-N1 [26] under the control of CMV promoter and the three genes were partially sequenced. For gene transfection, the cells were seeded at ∼90% confluence for 3 hrs before transfection with lipofectamine 2000 (Invitrogen, Paisley, UK) according to manufacturer's instructions as described previously [25]. The plasmid DNA of Pdx-1, NeuroD1 and Ngn3 were mixed in a ratio of 1∶3∶1 in weight prior to the transfection. The triple gene transfected hMSCs were then termed hMSC/PNNs. Empty plasmid–pIRES-N1 was used as a negative transfection control.

**Table 1:** Primer sets for cloning, PCR and sequence analyses.

Genes	Primer sets	Product length
Pdx-1 F1(Clone)	5′CGGGCCGCAGCCATGAACG3′	874 bp
Pdx-1 R1(clone)	5′CTCCTGCCTCTCATCGTGGTTCCTG3′	
Pdx-1 F2(PCR)	5′CCGCCGCCGCACCCGTTCC3′	508 bp
Pdx-1 R2(PCR)	5′CGACCCCGCCACCCCCGACAG3′	
Pdx-1 Seq. F	5′CAAAACCGCCGCATGAAGTGGA3′	
Pdx-1 Seq. R	5′AGCTGAGCCGGGAGGTGGTGGTGA3′	
NeuroD1 F1 (Clone)	5′AAATGAATTCATGACCAAATCGTACAGCGAGA3′	1093 bp
NeuroD1 R1 (Clone)	5′TCGCGTCGACCTAATCATGAAATATGGCATTGAGCTG3′	
NeuroD1 F2 (PCR)	5′ATGAACGCAGAGGAGGACTCACTG3′	410 bp
NeuroD1 R2 (PCR)	5′TTGGTGGTGGGTTGGGATAAGC3′	
Ngn3 F	5′GCGCCGGTAGAAAGGATGACGCC3′	677 bp
Ngn3 R (Clone and PCR)	5′CCCGGCTCCCTCCCTCTCCCTTAC3′	
INS F	5′CATCAAGCACATTGTCC3′	435 bp
INS R	5′CTGGTTCAAGGGCTTTATTC3′	
INS F (internal/PCR)	5 TCTACCTAGTGTGCGGGGAACGAG 3′	256 bp
INS R (internal/PCR)	5′CAGGAGGCGGCGGGTGTGG 3′	
Glucagon F	5′GCATTTACTTTGTGGCTGGGTC3′	420 bp
Glucagon R	5′AGTGATTTTGGTCTGAATC3′	
Somatostatin F	5′ GGCGCCGAGATGCTGTCCTG3′	314 bp
Somatostatin R	5′ TGCGTTCTCGGGGTGCCATAG3′	
Pancreatic polypeptide Y F	5′ CCACCTGCGTGGCTCTGTTACTAC3′	292 bp
Pancreatic polypeptide Y R	5′ CTGGGCTGGCGCTGCTCATGG3′	
GluT2 F	5′GTTTTGGGTGGTCCACTGGATG3′	523 bp
GluT2 R	5′GCCACAGATCATAATTGCCCAAG3′	
GLK F	5′GCCCCACAGCTCAACACAACCAG3′	597 bp
GLK R	5′CTCCCACCTTCACCAGCATCACC3′	
SUR1 F	5′CCGGGACGACAAGAGGACAGTGGT3′	401 bp
SUR1 R	5′GGATGCCGGCGGAGGACAGGTA3′	
Kir6.2 F	5′GGGGGCGCATGGTGACTGAG3′	506 bp
Kir6.2 R	5′GGTGCGGGCCTGGGTGGTGAT3′	
PC1/3 F	5′ GTCCTCTTTTGCGCT TGGTGTGC 3′	441 bp
PC1/3 R	5′ TCCTTTGCCCGTAATGCCTTTTTG 3′	
Actin F	5′CCCAGATCATGTTTGAGACC3′	664 bp
Actin R	5′CCAACAGGAGTACTTGCGCTCAG3′	

### Differentiation induction

To induce the hMSC/PNNs into a low-degree differentiation in vitro, the cells were converted into cluster culture in CMRL1066 medium (Invitrogen, Paisley, UK) overnight, 48 hrs after the triple gene transfection. The following morning, a cocktail of differentiation induction reagents was added to a final concentrations of 25pM activin A, 200pM betacellulin (R&D Systems, Oxford, UK), 10 mM nicotinamide (Sigma, Dorset, UK) and 16.5 mM glucose (termed ABNG) plus 10% FCS. The medium is replaced every 2 days with fresh ABNG. Cell samples were taken and assessed for expression of β-cell markers at the end of 6 days culture.

### Semi-quantitative RT-PCR

Total RNA was isolated using a RNA Miniprep Kit (Promega, Southampton, UK) and quantified using a spectrometer (Gene Quant II, Pharmacia Biotech) at 260 nm wavelength described previously [25], [27]. Briefly, 100 ng total RNA was converted into the 1^st^ strand cDNA with reverse transcriptase (Invitrogen, Paisley, UK) in a volume of 20 µl. The hot-start (Qiagen, Hilden, Germany) RT-PCR was performed with 95°C for 15 min for one cycle; then various amplification cycles for individual genes. Human β-actin gene was amplified in parallel as an internal control. The PCR primers are listed in Table 1. DNA relative quantity was analysed using a gel program (version 3.0 computer analyser of Media, Cybernetics, L.P, Silver Spring, MD) and expressed as relative to the density of β-actin gene PCR product.

### Electron Microscopy (EM)

Cells were fixed in 2.5% glutaraldehyde in 0.1 M phosphate buffer, followed by secondary fixation in 1% osmium tetroxide in 0.1 M phosphate buffer. The specimen was dehydrated through an ascending series of ethanols and embedded in TAAB epoxy resin (TAAB, Reading, UK). Sections, 80 nm thick, were cut on a Leica Ultracut E ultramicrotome (Leica, Milton Keynes, UK), stained with Uranyl acetate and lead citrate and examined by electron microscopy (Hitachi H7600, Berks, UK). For gold labelling experiments, staining with Uranyl acetate and lead citrate was omitted. The sections were incubated with monoclonal antibody against insulin (K36aC10 at 1∶100, Sigma, Dorset, UK) at 4°C overnight. After 3 washes with PBS, the sections were incubated with goat anti-mouse IgG (been pre-absorbed with human tissues) conjugated with 10 nm gold particle (EM.GMHL10, British Biocell, Cardiff, UK) and examined by electron microscopy (Hitachi H7600, Berks, UK). The negative control was performed in parallel with non-related IgG1 monoclonal antibody (replacing insulin antibody).

### Immunochemical/immunohistochemical staining

The methods have been described previously [25], [27]. Briefly, cultured cells or tissues were mounted with OCT compound (Merck ltd, Nottingham, UK) and cryosectioned at 5 µm intervals. The sections were incubated with antigen specific antibodies followed by fluorescence conjugated 2^nd^ antibodies and visualised under fluorescent or confocal microscopy. Two types of negative control were performed: one was by replacing the primary antibody with non-related subtype monoclonal antibodies or species related normal serum; the other was by staining the non-transplanted tissue sections with antigen specific antibodies. At least 3,000 cells per count and 3 counts per sample were performed.

### Animals and Surgical Procedures

Male SCID mice (20–25 g, C.B-17/Icr), purchased from Charles River UK Ltd, were selected as recipients for the manipulated hMSCs. The mice were maintained in filter-cages in the Comparative Biology Centre at King's College London, according to Home Office (UK) guidelines for Animal Scientific Procedures. Diabetes was induced by a single injection of STZ (180 mg/kg i.p) and confirmed by the presence of hyperglycaemia. The diabetic condition was allowed to stabilize for 3–10 days before transplantation. The diabetic mice were randomly allocated into transplant recipient and sham operated control groups. The transplantation procedures have been described previously [25], [27]. Briefly, under anaesthesia, a median lateral laparotomy was performed and the left kidney was exposed. Approximately 1,500 cell clusters (∼3×10^6^ cells) in a volume of 50 µl were transplanted into the kidney parenchyma by direct insertion of a Hamilton syringe into the kidney at the lower pole and the cells were discharged slowly into the parenchyma as the syringe was slowly withdrawn. In the sham control group, a cell-free saline injection was made in precisely similar fashion. Plasma glucose was measured using a G2 blood glucose sensor from MediSense (Abbott Laboratories, Abingdon, UK).

### Intra-peritoneal glucose tolerance test (IPGTT)

After the mice had been fasted over-night the basal plasma glucose level was determined. Glucose solution at 1.5 g/kg body weight was injected intraperitoneally and plasma glucose levels were determined at 30, 60, 90, 120 and 180 min post injection.

At the end of the experiments, blood, kidneys and pancreases were retrieved. Immunohistochemistry was performed to determine the phenotype of grafted cells and cell nuclei were counterstained with propidium iodide (Sigma, Dorset, UK) and analysed by fluorescent or confocal microscopy. Hormone-positive cells and the total transplanted cells (1500 graft cells per staining) were counted under fluorescence microscope and the percentage of positive cells was determined. The concentrations of human specific insulin and C-peptide were determined by immunoradioassay (DSL, Texas, USA). Organs from sham mice were studied in parallel as negative controls.

#### Antibodies

The monoclonal antibody to human insulin (clone K36aC10) and glucagon (clone K79bB10) were purchased from Sigma (Sigma, Dorset, UK). Rabbit anti human Ki67 antibodies were purchased from DAKO (DAKO, Glostrup, Denmark). Rabbit anti human somatostatin antibodies and PYY antibodies were from AbD Serotec (AbD Serotec, Oxford, UK). Goat polyclonal antibody to Pdx-1: (sc-14664), monoclonal antibody to NeuroD1: (sc-46684) and rabbit polyclonal antibodies to Neurogenin 3 (sc-25654) were purchased from Santa Cruz (Santa Cruz Biotechnology, CA, USA).

### Statistics

All data are expressed as mean+/− standard deviation. P<0.05 was considered as statistical different between groups, determined by ANOVAR.

## Results

### The cell renewal capacity and the expression of markers of both stem cell and pancreatic endocrine cells

Both cell lines (220R and 240L) showed the similar renewal capacity in monolayer culture (Fig. S1a). The doubling times were approximately 30 hrs, which did not change up to 10 cell passages (Fig. S1b). The cell number increased by approximately 15-folds per cell passage.

**Figure 1:**
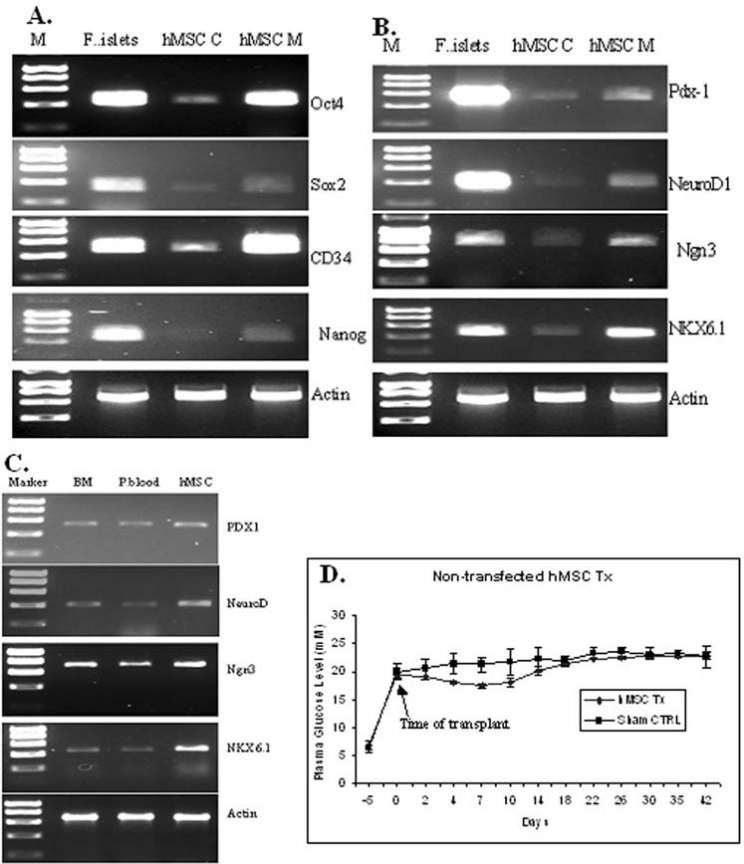
Characteristics of human marrow stromal cells (hMSCs). Panel 1a and 1b show the typical gel analyses of hMSCs (220R and 240L) in term of expression of makers for stem cells (1a) and pancreatic endocrine cells (1b); Lane M is the molecular weight, lane F, freshly isolated human islets, lane hMSC C is the hMSC in cluster culture and lane hMSC M is the hMSC in monolayer culture. Panel 1c shows the similarity in the expression of pancreatic endocrine cell marks among local generated primary human stromal cells and the 2 hMSCs lines: 220R and 240L. Lane BM is the primary stromal cells derived from bone marrow and Lane p blood is the primary stromal cells derived from peripheral blood. Panel 1d shows the potential of hMSCs to improve hyperglycaemia in the diabetic SCID mice. All experiments were repeated at least 3 times.

Both hMSC lines expressed the typical stem cell markers: CD34, Oct4, Sox2 and Nanog [28]–[34] (Fig. 1a) and pancreatic endocrine cell markers Pdx-1, NeuroD1, Ngn3 and Nkx6.1 [35]–[38] (Fig. 1b). Two additional hMSC primary cells generated from healthy donors in our institution, one from bone marrow and the other from peripheral blood, also expressed markers of endocrine cells observed in 220R and 240L (Fig. 1c), indicating that these properties represent a robust and reproducible hMSC phenotype. These cells were not analysed further.

On conversion to cluster culture (Fig. S1c), a condition favouring differentiation into the beta cell phenotype [27], the expression of CD34, Oct4, Sox2 and Nanog was greatly reduced (Fig. 1a). Unexpectedly, the expression of Pdx-1, NeuroD1, Ngn3 and Nkx6.1 was also reduced (Fig. 1b). The expression of Pdx-1, NeuroD1 and Ngn3 proteins was not detectable in the cluster cells by immunocytochemical staining (data not shown). The expression of markers of stem cells became undetectable in the implanted cells (Fig. S1d).

### Human MSCs secreted insulin *in vitro*, but it was not glucose regulated

Non-transfected hMSC (220R and 240L) lines were cultured in clusters in the presence of the differentiation inducers (ABNG) for 6 days. No insulin mRNA was detected in vitro by RT-PCR. Transplantation of these cells (3×10^6^ cells/mouse) into the kidney parenchyma of mice rendered diabetic by streptozotocin (STZ) treatment was associated with a short-lived small reduction in mouse plasma glucose (19.47+/−0.57 mM to 17.45+/−0.77 mM at day 7 post-transplantation p<0.01 n = 8) (Fig. 1d). Sham operated mice showed no change. The difference in plasma glucose levels between the two groups of mice at day 7 post-transplantation was significant (p<0.01), suggesting that the hMSCs might produce insulin *in vivo*, although this could not be confirmed by immunohistochemistry.

**Figure 2:**
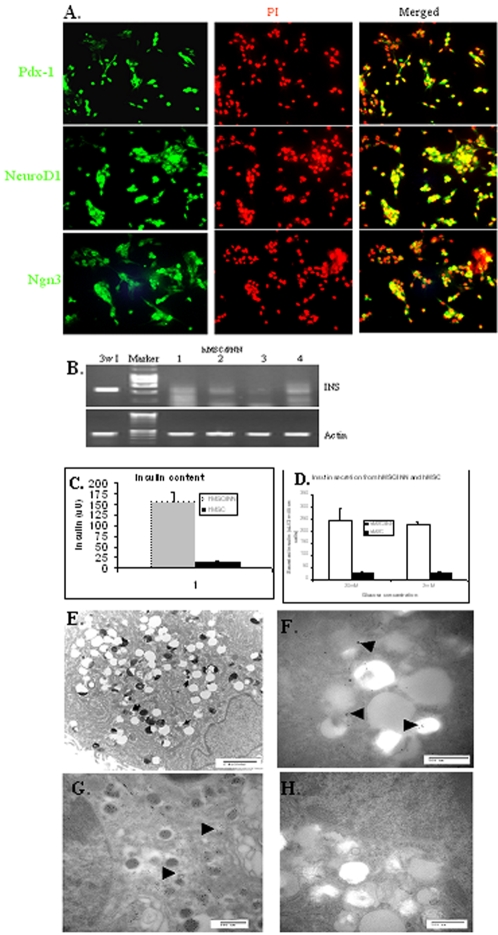
Human MSCs expressed insulin in vitro following differentiation induction. Panel 2a shows the expression of Pdx-1, NeuroD1 and Ngn3 in hMSCs 48 hr post transfection with the 3 genes. Majority of the gene product (green fluorescence) was located in nuclei, but some cells with cytoplasmic expression in all the 3 genes. Panel 2b shows the mRNA of preproinsulin in hMSC/PNNs following 6 days' differentiation induction in vitro, assessed by RT-PCR. The lanes 1–4 show the typical 4 different RT-PCR experiments on the expression of insulin in hMSC/PNNs, lane 3M shows the expression of insulin in human islet cells after 3 week in monolayer culture. Panel 2c shows the presence of insulin protein content in hMSC/PNNs following differentiation induction following correction with protein concentration. Panel 2d shows the insulin secretion by hMSCs and hMSC/PNNs following glucose challenge in a static incubation assay. Panel 2e shows the presence of insulin secretory granules in the hMSC/PNNs in a typical electronic microscopy picture and the magnification indicator is 2 microns. The typical insulin secretory granules are arrowed. Panel 2f shows the presence of insulin protein in hMSC/PNNs, as assessed by immunogold labelling experiments. Panel 2g shows the insulin protein in human islets, served as positive control of 2f, while 2h is the negative control of 2f, in which hMSC/PNNs were stained with non-insulin related IgG1 antibody. The black dots are the gold particles that labelled insulin protein. The magnification indicators in panel 2f–2h are 500 nm. All experiments were repeated at least 3 times.

One possible reason for the failure of the hMSCs to differentiate into a functional phenotype of beta cell is that the expression levels of Pdx-1, NeuroD1 and Ngn3 were too low. Human MSCs were transfected with a combination of Pdx-1, NeuroD1 and Ngn3 in a ratio of 1∶3∶1 in weight without subsequent selection and the transfectants are termed hMSC/PNNs. The transfection efficacy was aprox. 30% as assessed by counting the green fluorescent protein (GFP) positive cells under the fluorescent microscope against total cell number (Fig. S2a). Analyses of GFP expressing cells by fluorescence-activated cell sorter analysis, the transfection efficiency was >85% (Fig. S2b, S2c). The difference was possibly due to the different detection sensitivities in the two methods. The ratio of expression of the three genes in the transfected hMSCs was closed to 1∶3∶1 as assessed by RT-PCR as expected (Fig. S2d). The expression of the three genes was observed in majority of the cells 48 hr post transfection, as assessed by immunocytochemical staining. The 3 proteins were major located in nuclei, but some of them also presented in cytoplasm (Fig. 2a). For differentiation induction, cells were converted into clusters 48 hrs post-transfection, and incubated with differentiation reagents for 6 days. The hMSC/PNNs began to express low-level preproinsulin mRNA (Fig. 2b) and cellular insulin protein (156.25±21.17 µU/3×10^6^ cells) while non-transfected control cells did not (13.08±4.28 µU/3×10^6^ cells, p<0.01, Fig. 2c). The medium of the differentiated induced hMSC/PNNs at day 6 contained insulin (220±45.2 µU/3×10^6^ cells), which was not observed in the nontransfected hMSCs. To prove the true expression of insulin gene in vitro, the PCR DNA fragment corresponding with the expected size was cut out of the agarose gel following electrophoresis, purified and partially sequenced. The DNA sequence was matched to the authentic insulin DNA gene (NM_000207, data not shown). In addition, the purified DNA was used as a template to amplify the insulin gene using an internal insulin PCR primer set (Table 1) and the expected size of 256 bp PCR product was observed (data not shown). Intact hMSC/PNN cells secreted insulin (245±50.29 µU/3×10^6^ cells) at 20 mM glucose, ∼10-fold more than non-transfected hMSCs (24.65±6.21 µU/3×10^6^ cells, p<0.01). However, insulin secretion was not significantly different in hMSC/PNNs when glucose concentration was reduced to 2 mM (Fig. 2d). The expression of SUR1 and Kir6.2, a K^+^-ATP channel required for the regulation of insulin secretion, was not detectable in hMSC/PNNs in vitro (data not shown). Although immunocytochemical staining for insulin in the hMSC/PNNs for light microscopy did not provide conclusive evidences (data not shown), electron microscopy analyses did confirm the presence of insulin secretory granule-like structures in the hMSC/PNNs (Fig. 2e). The hMSC/PNNs picked up immuno-gold particles (Fig. 2f), as did the secretory granules of freshly isolated human islet cells (Fig. 2g). No gold particles were observed in the negative control staining, in which the hMSC/PNNs were stained with non-related IgG1 monoclonal antibody (Fig. 2h). The number of gold particles per granule was limited, probably as a result of the fixation process used (2.5% glutaraldehyde), which was selected to preserve granule ultrastructure but might impair the antigenicity of the insulin present.

### Human MSC/PNNs matured further in vivo

**Figure 3:**
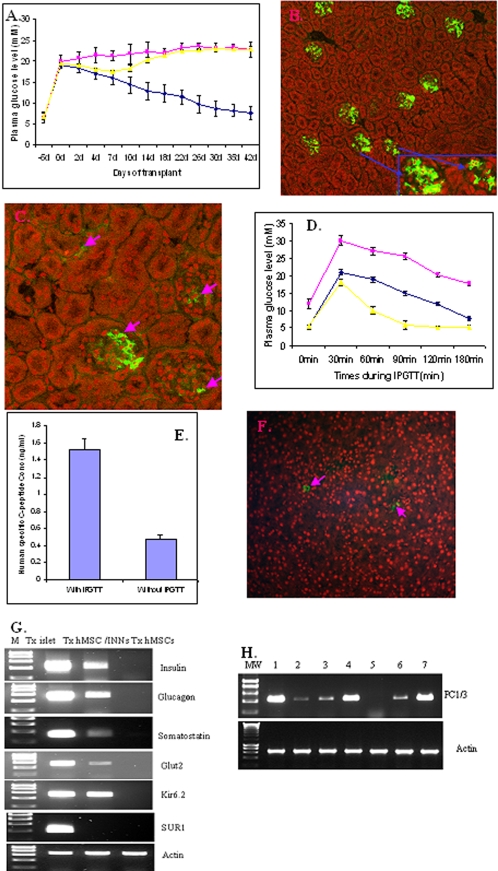
Effect of hMSC/PNNs on plasma glucose in diabetic mice. Panel 3a shows plasma glucose concentrations over days in transplanted mice. The symbol of blue diamond represents mice transplanted with hMSC/PNN (n = 16); The yellow triangle represents mice transplanted with hMSC with a phase of differentiation (n = 12) and the pink square represent the sham control (n = 12). Panel 3b and 3c show the presence of insulin and glucagon positive cells (green fluorescence) in the human MSC/PNNs transplanted grafts in typical mouse kidney sections respectively. In panel 3b, two insulin- expressing clusters were taken at higher power and shown in inset. Panel 3d shows the response of transplanted mice to a glucose challenge. The symbol of blue diamond represents mice transplanted with hMSC/PNN (n = 6); The pink square represents mice transplanted with the non-transfected hMSCs with a same phase of differentiation (n = 6) and symbol of yellow triangle represents mice transplanted with human islets (800 IEQ/mouse, n = 6). Panel 3e shows the differences in human specific C-peptide in mice after the glucose tolerant test (IPGTT) and without IPGTT. Panel 3f shows the typical residuals of the remaining insulin positive cells (green fluorescence) in the mouse panaceas of hMSC/PNNs recipients. Please note that the images is presented at low power to give a broad view of the tissue section, emphasizing the disappearing of islet cells from the STZ treated pancreases. Panel 3g shows the presences of mRNA of insulin, glucagons, somatostatin, GluT-2 and Kir6.2, but lacks of human SUR1 gene, in the kidneys transplanted with hMSC/PNN cells, using mouse kidneys transplanted with human islet cells as positive control, and mouse kidneys transplanted with hMSC cells as negative control. Panel 3h shows the expression of prohormone convertase 1/3 in hMSC/PNN cells, illustrating the effect of the 3 transpected genes on the expression of prohormone convertase 1/3. The sequences of the lanes are: MW–DNA molecular weight marker, 1–fresh human islet cells, 2–hMSC in monolayer, 3–hMSC in cluster culture, 4–hMSC/PNN cells with differentiation induction, 5–mouse kidney, 6-mouse kidneys transplanted with hMSC cells with a phase of differentiation induction and 7–mouse kidneys transplanted with hMSC/PNN cells with differentiation induction. All experiments were repeated >3 times.

To examine whether hMSC/PNNs would mature into glucose-responsive insulin-producing cells in vivo, hMSC/PNNs (3×10^6^ cell/mouse) were differentiation induced and then transplanted into the parenchyma of the left kidney in male SCID diabetic mice, using non-transfected hMSC cells as a control, which were differentiation induced in the same manner as for hMSC/PNNs. Mice transplantation with hMSC/PNNs was associated with a slow decline of initial hyperglycaemia (from 18.9+/−0.75 to 7.63+/−1.63 mM, p<0.01, n = 16). In 13 of the 16 mice, plasma glucose reached to normal ranges (6.9+/−0.64 mM) within 4 weeks, with normoglycaemia sustained for a further 2 weeks, at which time the animals were sacrificed (Fig. 3a). Immunohistochemical analyses on the kidneys bearing hMSC/PNNs showed the presence of insulin positive cells (12.5±2.6%, Fig. 3b, n = 10) and glucagon-expressing cells (6.43±1.94%, Fig. 3c, n = 10) among the grafted cells, while the negative controls on the consecutive sections did not show any positive staining, neither there were positive staining on non-transplanted kidney sections with the same anti-insulin antibody (Fig. S3). No somatostatin or pancreatic polypeptide-expressing cells were detected. Recipients of hMSC/PNNs responded positively to an intraperitoneum glucose tolerance test (IPGTT) by significantly lower plasma glucose within a 3 hr test period, although the hMSC/PNNs mice were less efficient than mice transplanted with fresh human islets (800 islet IEQ/mouse) (Fig. 3d). The mean of human specific C-peptide levels were approx. 1.5 ng/ml, a 3–fold higher in blood of the hMSC/PNN recipients after IPGTT than in non-IPGTT tested mice (Fig. 3e, p<0.01, n = 6). The pancreases from the hMSC/PNN mice showed only few individual insulin positive cells (Fig. 3f and Fig. S4).

RNA extracted from the hMSC/PNNs bearing kidneys showed the presence of insulin mRNA as an unique single band (Fig. 3g), which was dramatically different from the multiple banding pattern observed in the pre-transplant hMSC/PNNs (Fig. 2b), suggesting that the insulin-producing cells had undergone a phase of maturation and differentiation. The hMSC/PNNs engrafted kidneys also expressed glucagon and somatostatin mRNA (Fig. 3g), although somatostatin protein was not detectable in the grafts, confirming that the cells not only differentiated into beta cells but also into alpha cells and possibly somatostatin-producing cells as well. The hMSC/PNNs engrafted kidneys contained mRNAs for GluT-2 and Kir6.2 (Fig. 3g), key genes in beta cells for glucose intake and insulin secretion regulation. However, the expression of SUR1 gene was undetectable by RT-PCR (Fig. 3g). Control grafted kidneys from mice transplanted with non-transfected hMSCs cells did not show these changes (Fig. 3g). In addition, human MSCs express prohormone convertase 1/3 (NM_000439, PC1/3) (Fig. 3h lane 2) and the expression was enhanced when the cells were clustered and reached to similar expression level with fresh human islets when the cells were transfected with Pdx-1, NeuroD1 and Ngn3 and with differentiation induction (Fig. 3h lane 4), suggesting that these cells might be able to process proinsulin to mature insulin. The 3-gene transfection seemed to enhance the expression of PC1/3 in hMSC/PNNs as the different expression was observed in lanes between 3 and 5, 6 and 7 (Fig. 3h). Mouse kidney does not express the human version of the PC1/3 (Fig. 3h lane 5).

### Human MSCs did not form teratoma structure in the transplant recipients

**Figure 4:**
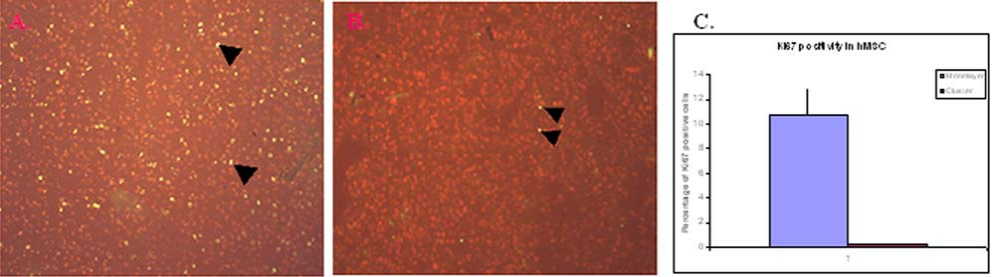
Analyses of the proliferation potential in hMSCs. Panel 4a shows the hMSC cells in monolayer culture, while the cluster cultured cells are shown in panel 4b. Panel 4c summarised the Ki67positive cells in both monolayer and cluster cultures. The yellow dots (the merged colour of green fluorescence and red propidium iodide) represent the Ki67positive cells. Some of the Ki67 positive cells are indicated with arrowhead. All experiments were repeated >3 times.

The potential risk of hMSCs to form teratoma in transplanted mice was ascertained by assessing the presence of proliferation marker Ki67 protein [39], in monolayer culture (Fig. 4a), in cluster culture in the presence of differentiation inducers ABNG (Fig. 4b) and in the mouse kidney sections containing the grafts of hMSC (in clusters). A mean of 10.79±2.14% in Ki67^+ve^ hMSCs after 5-days in monolayer culture was observed, which fell to 0.18±0.048% (p<0.01) in cluster culture with differentiation inducers (Fig. 4c) and beyond detection in the implanted cells. Neither Ki67+ve cells nor teratoma structures were observed in the grafts 1–6 weeks post-transplantation, although it should be noted that no study lasted >6 weeks post transplantation.

## Discussion

This study explores the potential of human MSCs as a source for glucose-regulated insulin-producing cells and also addresses some of the safety issue of these cells for clinical use.

We developed a robust system to direct the differentiation of hMSCs into insulin-producing cells through a transient, non-viral genomic reprogramming approach. The half-life of the transfected plasmid DNA was 8ds in cluster cells, while it was 3 days in monolayer cells. This is possibly due to the near zero proliferation activity in the clustered cells (Zhao and Huang, unpublished). Although hMSC have previously been shown to express insulin at mRNA level with the infection of recombinant Pdx-1 adenovirus [5], only a small number of MSCs became insulin positive cells when no gene modification had taken place [20]–[22]. A recent study showed that genetically modulated hMSCs with recombinant Pdx-1 adenovirus were able to reduce mouse blood glucose with detectable human C-peptide (0.06–0.39 ng/ml) [23]. Our approach using a non-virus triple gene transfection approach has resulted in a much higher percentage of insulin expressing cells (>12%). The mean human C-peptide in the non-glucose tolerant test mice was ∼0.5 ng/ml (n = 6). In addition, there was significant number of glucagon-expressing cells (>6%). The majority of hMSC/PNNs transplanted mice (13/16) returned to normoglycaemia. The presence of mRNAs for insulin, glucagon, somatostatin, GluT-2 and Kir6.2 in the cells from the rescued kidneys shows that the cells have developed into a phenotype of pancreatic endocrine cells. The presence of insulin mRNA in the graft bearing kidneys refutes the possibility that the insulin in the insulin producing cells is derived from the deposition of circulating insulin [40].

Human MSCs have a high renewal capacity and express a typical panel of stem cells markers: CD34, Oct4, Sox2 and Nanog. Cells from human pancreases were recently shown to contain cells expressing these markers of stem cells [41]. The proliferation rate for hMSCs was not changed up to 10 passages in both 220R and 240L hMSC lines (Fig. S1b). Potentially hMSCs could become almost unlimited cell sources as the cells number increased ∼15 folds per cell passage. For example, if we started with one million cells, there would be >10^16^ cells after 10 cell passages. The other advantage in hMSCs is that the proliferation capacity reduces dramatically following the differentiation induction phase and ceases to proliferate following transplantation as no Ki67 protein was detected from the grafts, which is different from the embryonic stem cells that form teratoma in recipients [42]. More interestingly the cells also express a panel of key genes required for the development and maintenance of functional phenotypes of pancreatic beta cells: Pdx-1, NeuroD1, Ngn3 and Nkx6.1. The expression of Pdx-1, NeuroD1 and Ngn3 in hMSC was a novel observation. Based on the assumption that MSCs could differentiate into neurons because they have expressed key neuronal genes [15], hMSCs may be taken to have innate potential to become beta cells, since they express these key beta cell markers (Pdx-1, NeuroD1 Ngn3, and Nkx6.1). These properties of hMSCs seem to represent a robust and reproducible hMSCs phenotype, as the expression of the same markers for stem cell and endocrine cells was detected in two additional hMSC primary cells derived from healthy donors in our own institution (Fig. 1e). Despite this potential, intact hMSCs seemed unable to differentiate into insulin-producing cells in vivo, in line with others' observations [43]–[45]. We believe that single Pdx-1 gene is not sufficient to generate functional insulin-producing cells [25]. The rationale of using Pdx-1, NeuroD1 and Ngn3 as genomic modulators is that Pdx-1, NeuroD1 and Ngn3 genes are all required for the development of beta cells, while Pdx-1 and NeuroD1 play important roles in the maintenance for the functional phenotypes of beta cells. As hMSCs are not embryonic cells, we are not trying to mimic the patterns in the beta cell development. We simply hypothesised that the combination of these three genes would work synergistically to make hMSCs respond to the cocktail of differentiation inducers and to initiate the differentiation in vitro. We also hypothesised that the in vitro induced cells would differentiate further into glucose responsive insulin producing cells under the hyperglycaemia with right supporting environments in the SCID mice, based on our previous observation [25], [27]. It has been shown that Ngn3 can initiate the differentiation of cells toward endocrine cells by modulating the E-box in the promoter of NeuroD1 gene [46]. There were reports that Pdx-1 works better together with Ngn3 or NeuoD1 to enhance the insulin expression [47]. Indeed the combination of Pdx-1, NeuroD1 and Ngn3 achieved more insulin-producing cells than the combination of Pdx-1, NeuoD1 and Isl1 (data not shown). The gene ratio was created by mimicking the mRNA levels of the three genes in the freshly isolated human islets and was established as the best combination following a serial of tests.

At least in vitro, the amount of insulin content and in secretion was fairly small, estimated as 1/1000–1/500 respectively of freshly isolated human islets. The insulin secretion was not glucose regulated as components of K^+^-ATP channel was not detected. Insulin was synthesized and secreted at the same time. This may explain why relatively lower insulin content (156 µU/3×10^6^ cells) than insulin secreted into the differentiation induction medium (220±45.2 µU/3×10^6^ cells). The cells may not establish a storage capacity for the synthesized insulin in vitro. This phenomenon is similar to those described for persistent hyperinsulinaemic hypoglycaemia of infancy (PHHI) cells. In PHHI cells the K^+^-ATP channel is mutated and the insulin is secreted as it is synthesized [48]. Nevertheless, our cells did differentiate further following transplantation and developed an element of glucose-responsive insulin secretion in vivo, as the plasma glucose level was reduced in association with the elevated levels in both insulin and C-peptide after a glucose challenge. These cells had high-level expression of Kir6.2, which was not present in vitro. There was no expression of SUR1 gene in vitro or in vivo. Whether the cells developed an alternative mechanism to regulate insulin secretion in these cells is not clear. Mice lack functional SUR1 protein appeared only glucose tolerant impairment but not diabetes at least in some period of their life [49], suggesting that beta cells may be able to compensate the lost function of K-ATP channel. It may be relevant that the insulin-producing cells were present in clusters similar to those seen in grafts of human islet cells, and distinct from our previous study in which insulin-producing cells were scattered, through a single Pdx-1 gene transfection [25]. There is evidence that beta cells function better in cluster structures [50], and forming clusters may be integral to their nature. The cluster formation suggests that the present triple gene transfection may be superior to single Pdx-1 gene transfection.

How the cells developed into a functional phenotype in vivo is not clear and is worthy of further exploration. This study provides a model to modify hMSCs further for glucose-regulated-insulin-producing cells for the clinical therapy of TIDM. It offers the potential for generating new islet cells from patients' own bone marrow, although further manipulation is likely to be required to achieve cells resistant to inflammatory damage and immunological attack. The interpretation of this study for clinical use is limited as hMSC lines were used rather than primary hMSC cells.

## Supporting Information

Figure S1 Characterization of human MSC cells. Panel 1a and 1c show the morphologies of hMSCs in monolayer and in cluster respectively. Panel 1b shows the proliferation capacities of cells at passages between 2 and 10. Panel 1d shows the typical gel analyses of hMSCs in term of expression of makers for stem cells before and after the differentiation induction. Lanes 1, 3, 5,7 and 9 are the hMSCs before the implantation and Lanes 2, 4, 6, 8 and 10 are the hMSCs after the implantation.
(0.30 MB TIF)

Figure S2 Assessment of the transfection of hMSC cells. Panel 2a shows the transfection efficiency in hMSC cells. The cells that have been transfected with the pIRES2-EGFP plasmid (BD Bioscience Clontech, Oxford UK) show the expression of green fluorescence protein (GFP). The transfection was performed with pIRES2-EGFP plasmid using lipofectamine 2000 reagents (Invitrogen, Paisley, UK). Approx. 30±3.56% cells were positive for GFP by manual cell counting. Panel 2b and 2c show the GFP positive hMSC cells assessed by fluorescence-activated cell sorter analysis according to method published previously [51]. Over 85% cells were shown positive for GFP (2b) against the negative control (2c), transfected with non-GFP plasmid. Panel d shows the typical PCR analyses of the expression of Pdx-1, NeuroD1 and Ngn3 Pre- and 72 hr post-transfection with the three genes.
(0.39 MB TIF)

Figure S3 Assessment of the specificity of anti human insulin antibody. Non-transplanted SCID mouse kidney cryosections were used to assess the specificity of anti human insulin monoclonal antibody (K36aC10, Sigma, Dorset, UK) in parallel with hMSC/PNN transplanted mouse kidney sections. The typical fluorescent microscopy image shows that there is no specific binding of this antibody to normal SCID mouse kidney tissues under the titration used in this study.
(2.54 MB TIF)

Figure S4 Assessment the insulin expressing cells in mouse pancreases following streptozotocin treatment. Mouse pancreases retrieved from the hMSC/PNN-transplanted mice were cryosectioned and stained for insulin (green). The cell nuclei were counterstained with propidium iodide (red). The images were taken at low power to emphasize that only very few insulin-expressing cells were present in the moue pancreases. No intact islets were observed in the mouse pancreases.
(1.86 MB TIF)
