# Supplementary figures and images for: Amelioration of Streptozotocin-Induced Diabetes in Mice with Cells Derived from Human Marrow Stromal Cells
Source: PLoS One. 2008 Jul 16;3(7):e2666. doi: 10.1371/journal.pone.0002666 (PMC2441861; doi:10.1371/journal.pone.0002666)

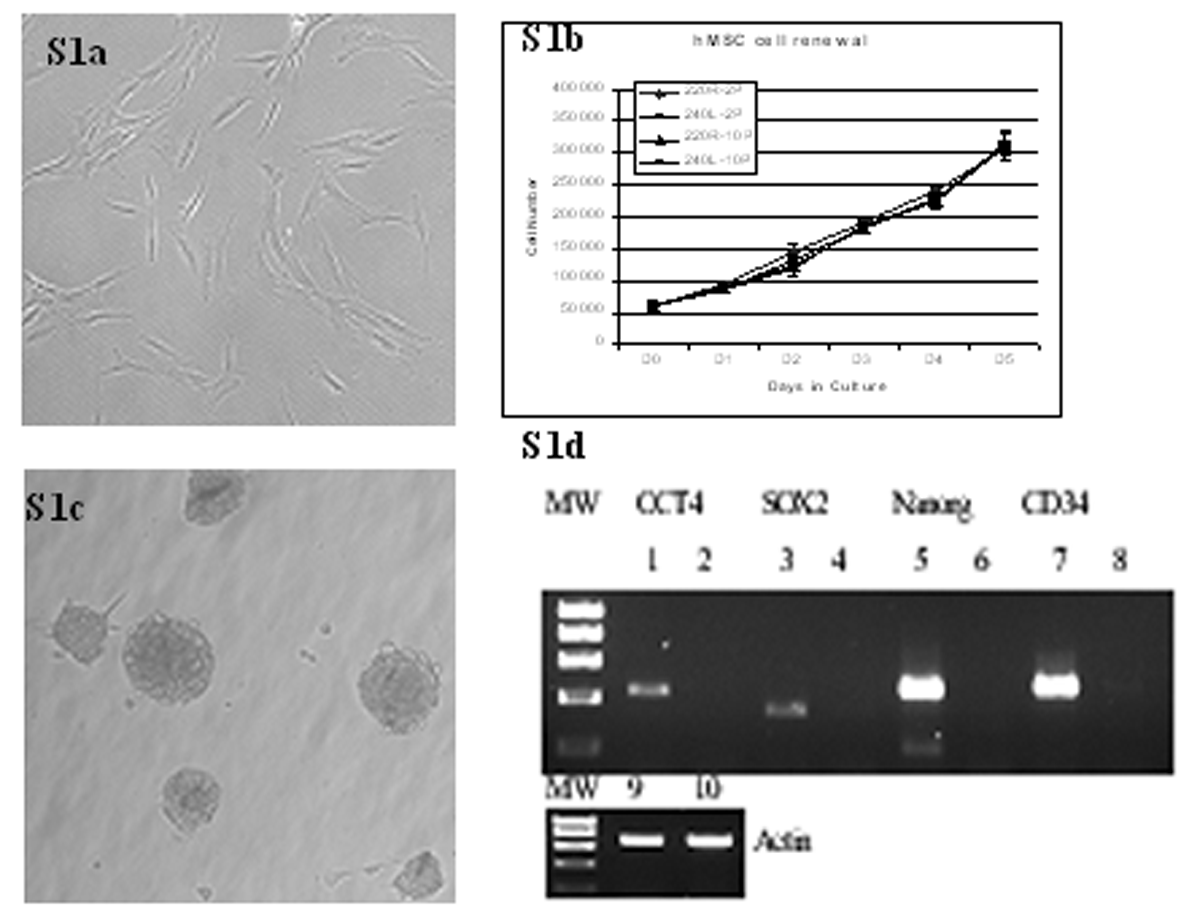

Supplement: Figure S1 — Characterization of human MSC cells. Panel 1a and 1c show the morphologies of hMSCs in monolayer and in cluster respectively. Panel 1b shows the proliferation capacities of cells at passages between 2 and 10. Panel 1d shows the typical gel analyses of hMSCs in term of expression of makers for stem cells before and after the differentiation induction. Lanes 1, 3, 5,7 and 9 are the hMSCs before the implantation and Lanes 2, 4, 6, 8 and 10 are the hMSCs after the implantation. (0.30 MB TIF) [file pone.0002666.s001.tif]

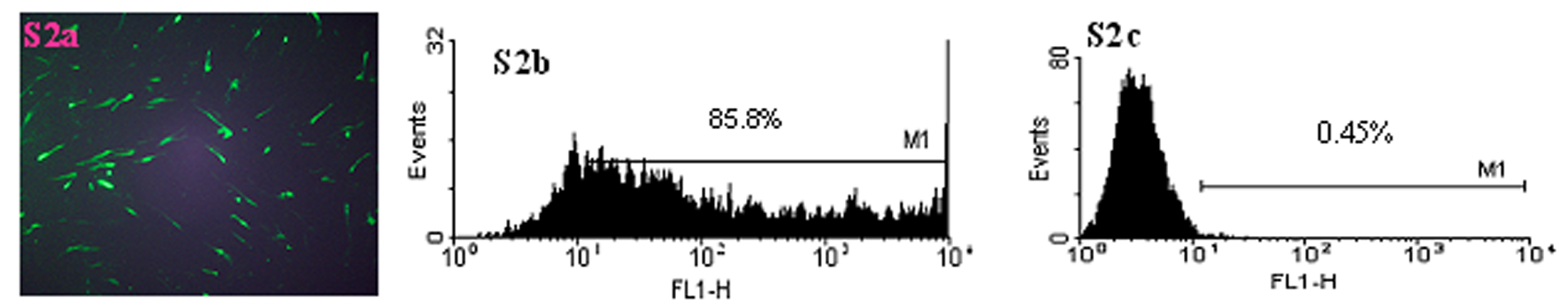

Supplement: Figure S2 — Assessment of the transfection of hMSC cells. Panel 2a shows the transfection efficiency in hMSC cells. The cells that have been transfected with the pIRES2-EGFP plasmid (BD Bioscience Clontech, Oxford UK) show the expression of green fluorescence protein (GFP). The transfection was performed with pIRES2-EGFP plasmid using lipofectamine 2000 reagents (Invitrogen, Paisley, UK). Approx. 30±3.56% cells were positive for GFP by manual cell counting. Panel 2b and 2c show the GFP positive hMSC cells assessed by fluorescence-activated cell sorter analysis according to method published previously [51]. Over 85% cells were shown positive for GFP (2b) against the negative control (2c), transfected with non-GFP plasmid. Panel d shows the typical PCR analyses of the expression of Pdx-1, NeuroD1 and Ngn3 Pre- and 72 hr post-transfection with the three genes. (0.39 MB TIF) [file pone.0002666.s002.tif]

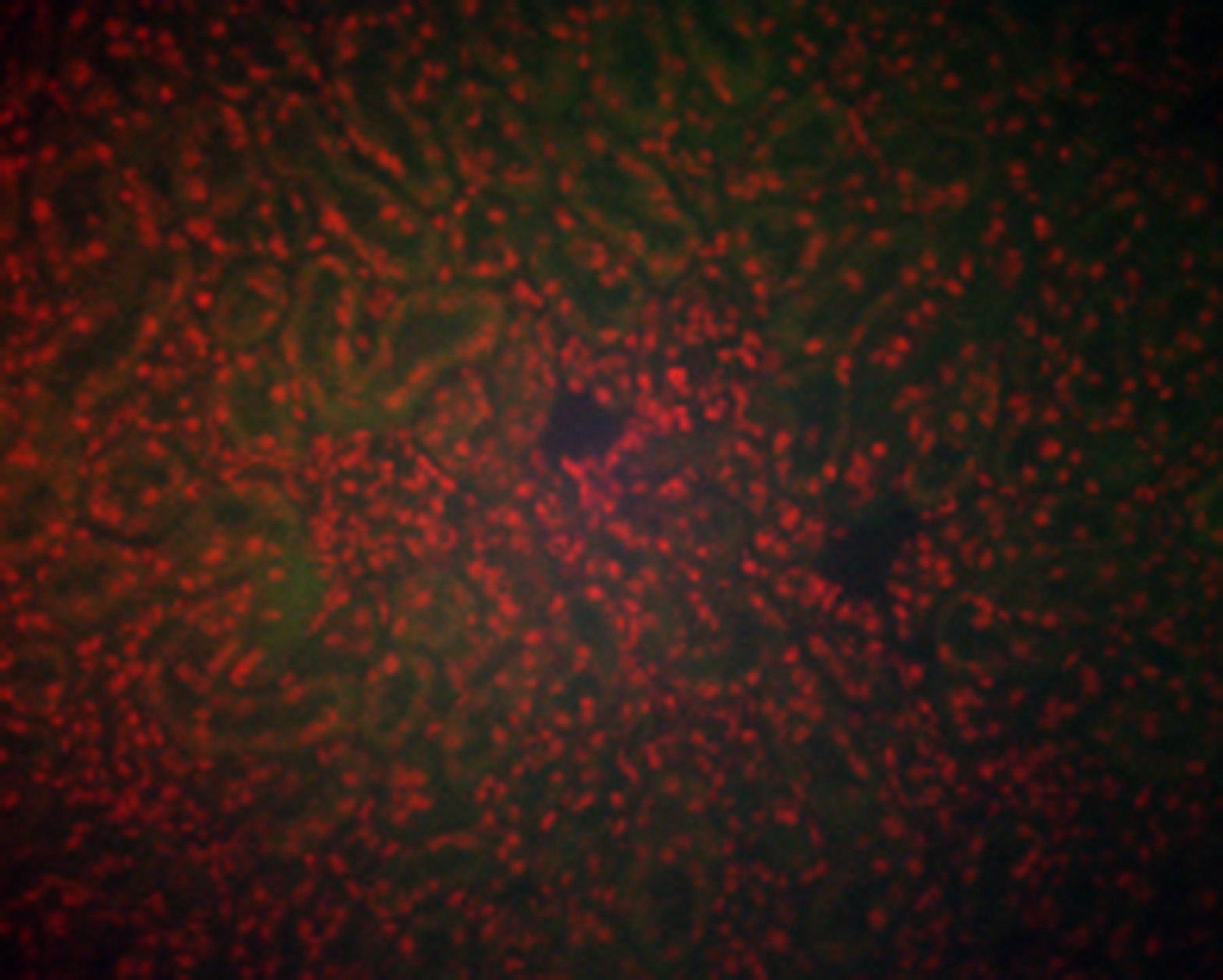

Supplement: Figure S3 — Assessment of the specificity of anti human insulin antibody. Non-transplanted SCID mouse kidney cryosections were used to assess the specificity of anti human insulin monoclonal antibody (K36aC10, Sigma, Dorset, UK) in parallel with hMSC/PNN transplanted mouse kidney sections. The typical fluorescent microscopy image shows that there is no specific binding of this antibody to normal SCID mouse kidney tissues under the titration used in this study. (2.54 MB TIF) [file pone.0002666.s003.tif]

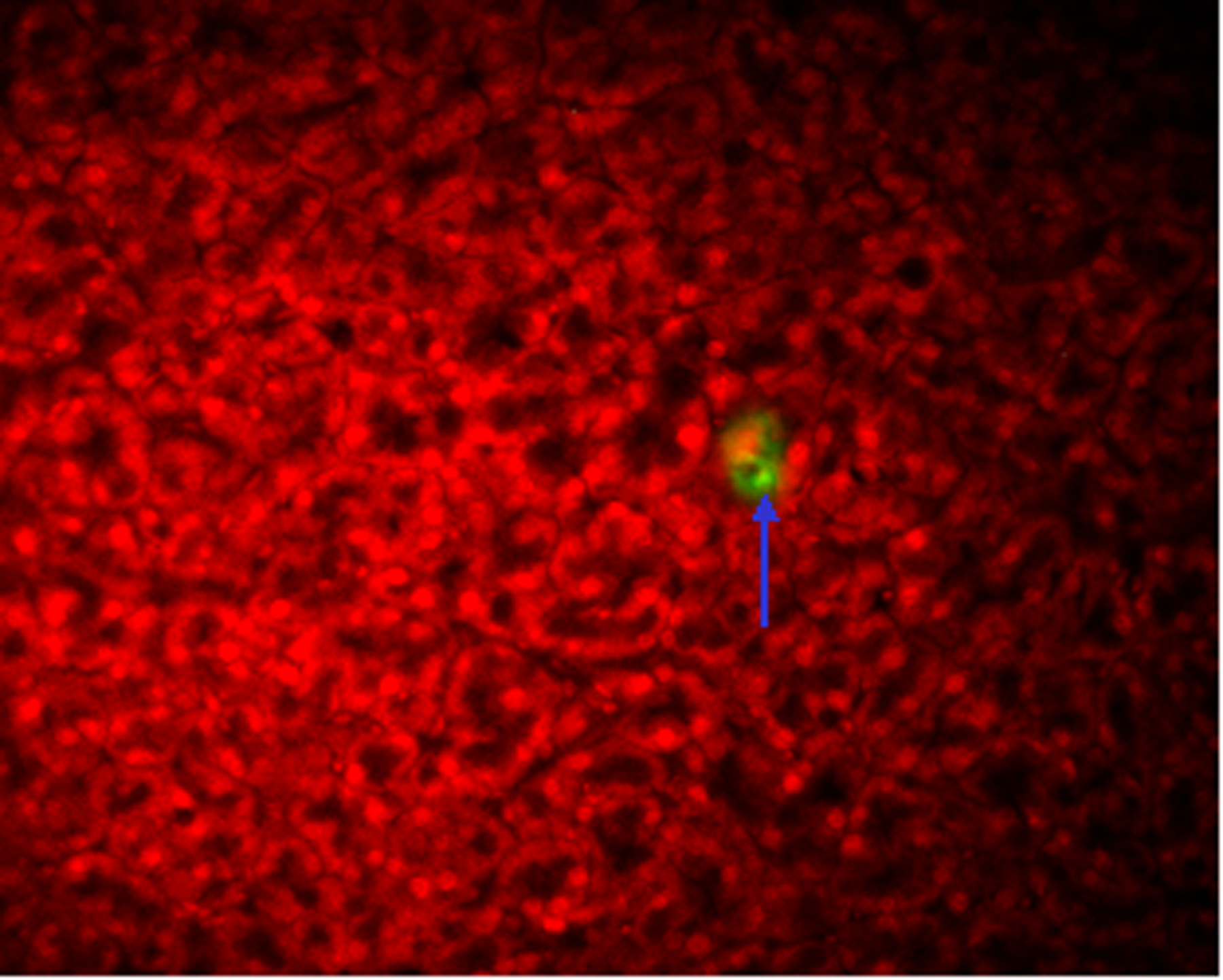

Supplement: Figure S4 — Assessment the insulin expressing cells in mouse pancreases following streptozotocin treatment. Mouse pancreases retrieved from the hMSC/PNN-transplanted mice were cryosectioned and stained for insulin (green). The cell nuclei were counterstained with propidium iodide (red). The images were taken at low power to emphasize that only very few insulin-expressing cells were present in the moue pancreases. No intact islets were observed in the mouse pancreases. (1.86 MB TIF) [file pone.0002666.s004.tif]
